# In Vitro Assessment of Cortisol Release Inhibition, Bioaccessibility and Bioavailability of a Chemically Characterized *Scutellaria lateriflora* L. Hydroethanolic Extract

**DOI:** 10.3390/molecules29030586

**Published:** 2024-01-25

**Authors:** Daniele Giuseppe Buccato, Hammad Ullah, Lorenza Francesca De Lellis, Roberto Piccinocchi, Alessandra Baldi, Xiang Xiao, Carla Renata Arciola, Alessandro Di Minno, Maria Daglia

**Affiliations:** 1 Department of Pharmacy, University of Napoli Federico II, Via D. Montesano 49, 80131 Naples, Italy; danielegiuseppe.buccato@unina.it (D.G.B.); hammad.ullah@unina.it (H.U.); lo.delellis2@libero.it (L.F.D.L.); alessandra.baldi.alimenti@gmail.com (A.B.); 2 Level 1 Medical Director Anaesthesia and Resuscitation A. U. O. Luigi Vanvitelli, Via Santa Maria di Costantinopoli, 80138 Naples, Italy; roberto.piccinocchi@policliniconapoli.it; 3 School of Food and Biological Engineering, Jiangsu University, Zhenjiang 212013, China; xiaoxiang1@aliyun.com; 4 Laboratory of Immunorheumatology and Regenerative Medicine, Laboratory of Pathology of Implant Infections, IRCCS Istituto Ortopedico Rizzoli, Via di Barbiano 1/10, 40136 Bologna, Italy; carlarenata.arciola@ior.it; 5 Department of Medical and Surgical Sciences (DIMEC), University of Bologna, Via San Giacomo 14, 40126 Bologna, Italy; 6 CEINGE-BiotecnologieAvanzate, Via Gaetano Salvatore 486, 80145 Naples, Italy; 7 International Research Center for Food Nutrition and Safety, Jiangsu University, Zhenjiang 212013, China

**Keywords:** *Scutellaria lateriflora* L., cortisol, stress, bioaccessibility, bioavailability, osteoporosis

## Abstract

Excess cortisol release is associated with numerous health concerns, including psychiatric issues (i.e., anxiety, insomnia, and depression) and nonpsychiatric issues (i.e., osteoporosis). The aim of this study was to assess the in vitro inhibition of cortisol release, bioaccessibility, and bioavailability exerted by a chemically characterized *Scutellaria lateriflora* L. extract (SLE). The treatment of H295R cells with SLE at increasing, noncytotoxic, concentrations (5–30 ng/mL) showed significant inhibition of cortisol release ranging from 58 to 91%. The in vitro simulated gastric, duodenal, and gastroduodenal digestions, induced statistically significant reductions (*p* < 0.0001) in the bioactive polyphenolic compounds that most represented SLE. Bioavailability studies on duodenal digested SLE, using Caco-2 cells grown on transwell inserts and a parallel artificial membrane permeability assay, indicated oroxylin A glucuronide and oroxylin A were the only bioactive compounds able to cross the Caco-2 cell membrane and the artificial lipid membrane, respectively. The results suggest possible applications of SLE as a food supplement ingredient against cortisol-mediated stress response and the use of gastroresistant oral dosage forms to partially prevent the degradation of SLE bioactive compounds. In vivo studies and clinical trials remain necessary to draw a conclusion on the efficacy and tolerability of this plant extract.

## 1. Introduction

*Scutellaria lateriflora*, commonly known as American skullcap, is one of the most widely utilized nerve tonics in Western herbal medicine. For over 200 years, *S. lateriflora* has been used for the treatment of anxiety and the promotion of a healthy menstrual cycle. Other traditional uses of skullcap include hysteria, delirium tremens, withdrawal from barbiturates and tranquilizers, seizures, bronchitis, dysentery, diarrhea, jaundice, hepatitis, tumors, hypertension, and thrombosis [1]. Chemically, the aerial parts of this plant contain various classes of polyphenols, essential oils, diterpenoids, and amino acids. The polyphenolic profile of the hydroalcoholic extract used in this study, obtained from the aerial parts of the plant *S. lateriflora*, was previously determined using ultra-high-performance liquid chromatography coupled with a Q-Exactive hybrid quadrupole orbitrap mass spectrometer. This analysis allowed for the identification of over one hundred compounds, including flavonoids (baicalein, baicalin, dihydrobaicalin, oroxylin A, wogonin, lateriflorein, lateriflorin, scutellarin, ikonnikoside I, oroxylin A-7-*O*-glucuronide, and 2′-methoxy-chrysin-7-*O*-glucuronide), and phenolic acids (cinnamic acid, caffeic acid, ferulic acid, and *p*-coumaric acid) [2,3].

The Italian Health Ministry guidelines on the traditional effects of vegetable extracts used in food supplements report that *S. lateriflora* shows relaxation activity, but the scientific literature only reports studies on the antistress effects of another species of the genus Scutellaria, *Scutellaria baicalensis*, while studies on *S. lateriflora* are absent. Udintsev et al. [4] demonstrated a normalization of all hormonal metabolic alterations, including levels of insulin, glucose, urea, corticotropin, and hydroxycorticosteroids, in rats exposed to stress by fixation. Ryu et al. [5] and Lee et al. [6] reported the antistress effects of *S. baicalensis* extract in rodent experimental models, where the extract showed a considerable decrease in the blood levels of corticosterone.

Cortisol, a stress hormone, is released by the hypothalamic-pituitary-adrenal (HPA) axis and acutely and chronically affects the metabolic, cardiovascular, and nervous systems. Thus, a balanced regulation of the release of stress-induced cortisol is essential to maintaining homeostasis and a healthy status [7]. The amygdala sends signals to the hypothalamus in response to stress, and this activates the sympathetic nervous system, resulting in the release of catecholamines and cortisol by the adrenal glands. Catecholamines, such as adrenaline, increase heart and respiratory rates, while cortisol provides energy to the body and allows the body to stay on high alert in response to stress [8,9]. Cortisol release is regulated by a negative feedback mechanism in the central nervous system, where basal nonstressed cortisol secretion follows the circadian rhythm, with levels in the body rising sharply upon awakening and declining over the course of the day [10].

Dysregulated and excessive secretion of cortisol is associated with psychiatric issues, mainly insomnia, anxiety, and depression. Literature data have shown a significant relationship between sleep deprivation and HPA axis hyperactivation, resulting in neuroendocrine dysregulation [11]. One study reports a positive correlation between polysomnographic indices of insomnia in adults and urinary levels of free cortisol [12]. Insomnia patients usually present with high cortisol levels, mainly in the evening and at the onset of sleep, suggesting that hypercortisolism could be a marker of corticotropin-releasing hormone and noradrenaline activity during the night [13]. Importantly, HPA axis-induced sleep fragmentation and the associated increase in cortisol levels suggest that the HPA axis contributes not only to the establishment, but also to the continuation of chronic insomnia [14]. Moreover, approximately 50% of newly diagnosed patients with depression present elevated levels of cortisol [15]. In addition, increased levels of cortisol in the body may also contribute to osteopenia and osteoporosis by decreasing bone mineral density. It alters bone turnover, impairs intestinal absorption and renal reabsorption of calcium, and inhibits reproductive hormones in postmenopausal women [16].

There are a number of drugs that have long been proven to alter cortisol synthesis and/or release, including serotonin antagonists, dopaminergic agonists, metyrapone, ketoconazole, fluconazole, reserpine, valproic acid, and somatostatin analogs. However, these drugs present questionable efficacy and tolerability and are only recommended for severe forms of hypercortisolism, such as Cushing syndrome [17,18]. Patients suffering from insomnia, anxiety, and mild-to-moderate depression, which are not eligible for the available anticortisol therapies, are usually treated with conventional drugs targeting the symptoms of the disease. Thus, there is a strong need for the development of new alternative agents with cortisol inhibitory properties and favorable safety profiles, which can then be used in the treatment of mild-to-moderate hypercortisolism, such as sleep disorders, stress, and anxiety.

The present study is aimed at an in vitro evaluation of cortisol inhibition activities as a possible mechanism of action of a chemically characterized *S. lateriflora* extract. In view of its use as a food supplement ingredient, the in vitro bioaccessibility and bioavailability of the extract were also studied.

## 2. Results

### 2.1. Bioaccessibility of S. lateriflora Extract after In Vitro Simulated Gastric, Duodenal, and Gastroduodenal Digestion

**Figure 1:**
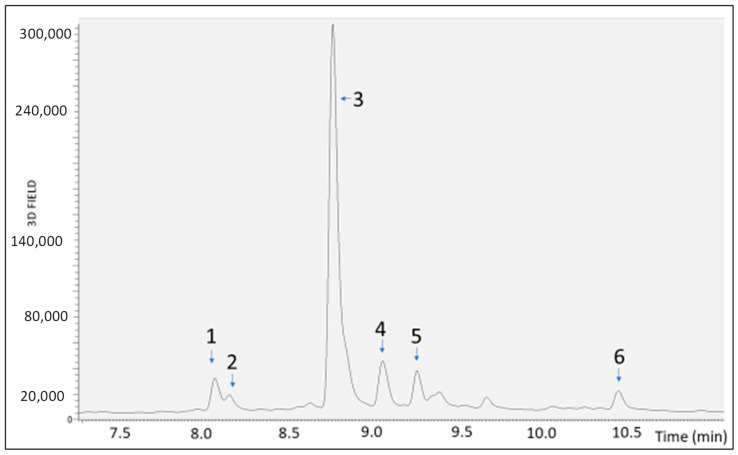
PDA chromatograms of *S. lateriflora* extract not submitted to digestion, obtained by RP-UHPLC-PDA-ESI-MS/MS analysis. 1: apigenin derivative, 2: scutellarin, 3: baicalein 6-glucuronide, 4: naringenin 7-*O*-d-hexoside 6″ acetate, 5: oroxilyn A glucuronide, and 6: genistein.

In view of the development of a food supplement based on *S. lateriflora* extract, the extract, whose metabolic profiling was previously determined using UHPLC-MS, was submitted to gastric, duodenal, and gastroduodenal digestion to evaluate its bioaccessibility. Then, the extract was analyzed using RP-UHPLC-PDA-ESI-MS/MS before (Figure 1) and after in vitro simulated digestion. The results show that the gastric, duodenal, and gastroduodenal digestion processes induced a statistically significant reduction (*p* < 0.0001) in the peak area of the most representative compounds (i.e., apigenin derivative, scutellarin, baicalein 6-glucuronide, naringenin 7-*O*-d-hexoside 6″ acetate, oroxilyn A glucuronide, and genistein), as compared to the undigested extract, revealing a moderate to high degradation. In fact, as shown in Table 1, the gastroduodenal digestion process resulted in the degradation of scutellarin (61.6%) and genistein (73.2%) to a greater extent than the other compounds, for which the percentage reduction in the chromatographic peak area ranged from 20.7% for oroxylin A glucuronide after duodenal digestion to 53.2% for the apigenin derivative after the gastroduodenal digestion process.

### 2.2. Bioavailability of S. lateriflora Extract

Considering the degradation of the phytocomplex after gastric digestion, the use of *S. lateriflora* extract as a food supplement ingredient, and the recommendation to use gastroresistant dosage forms, the bioavailability of *S. lateriflora* extract was assessed for the duodenal digested sample, aiming to mimic the conditions of the release of the phytochemical from gastroresistant dosage forms.

#### 2.2.1. Evaluation of Viability of Caco-2 Cells after Treatment with Duodenal Digested *S. lateriflora* Extract

**Figure 2:**
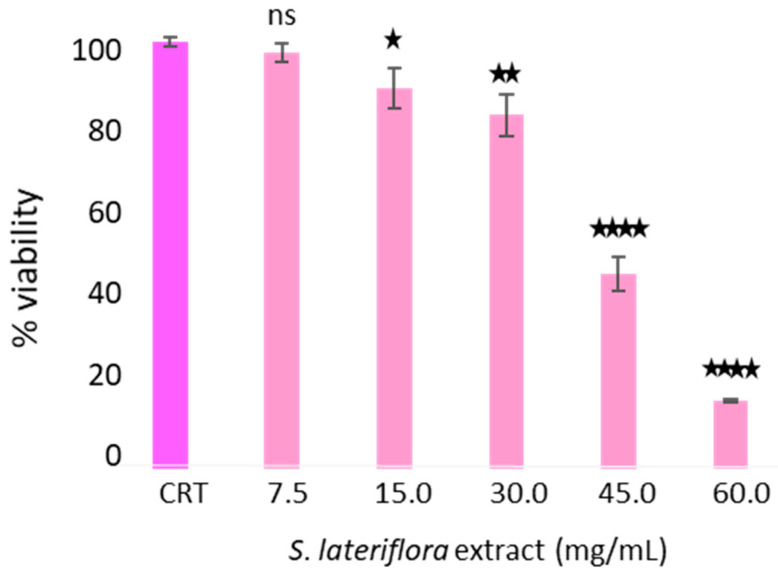
Caco-2 cell viability after treatment with the duodenal-digested *S. lateriflora* extract at a concentration range of 60–7.5 mg/mL and a control group (CTR). Viability values are calculated as averages from three independent assays, and the standard deviation is provided. The CTR represents cells not treated with *S. lateriflora* extract, set at 100% viability. Statistical significance is denoted as follows: * *p* < 0.05, ** *p* < 0.01, **** *p* < 0.0001, and ns: not significant.

To determine the maximum noncytotoxic concentration of duodenal digested *S. lateriflora* extract on Caco-2 cells grown on transwell inserts used as an absorption model, the cytotoxicity of the duodenal digested extract was evaluated using an MTT assay. As shown in Figure 2, at concentrations of 60.0–45.0 mg/mL, the extract caused a statistically significant decrease (*p* < 0.0001) in cell viability percentage in comparison to the control sample (cells not treated with the duodenal digested *S. lateriflora* extract). The extract concentration of 30 mg/mL was used in the bioavailability assay, as at this concentration the cell viability percentage was found to exceed 70%.

#### 2.2.2. Evaluation of Transepithelial Electrical Resistance (TEER) and Tight Junction ZO-1 Protein

To evaluate the integrity of the Caco-2 cell monolayer before (S1, S2, S3) and after cell incubation with the duodenal digested *S. lateriflora* extract (SD1, SD2, SD3), the TEER values were determined. The results show that the monolayer remained intact, since the recorded TEER values (1656 ± 12.8 Ω cm^2^) remained higher than 300 W cm^2^, a value considered the minimum acceptable transendothelial electrical resistance (Table 2).

**Figure 3:**
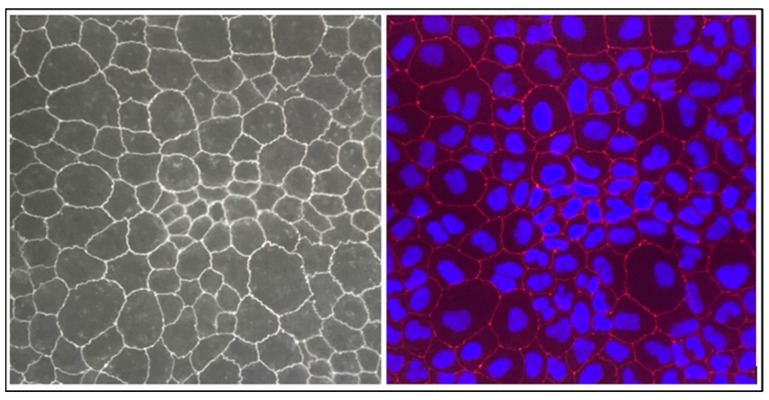
Morphology of Caco-2 cell monolayers. Fluorescent microscope image showing the top view of a Caco-2 monolayer, where the cells’ borders can be distinguished by immunocytochemical staining of the tight junction protein ZO-1 (red) and DAPI counterstaining for nuclei identification (blue).

To better understand the features of the Caco-2 cell monolayer, an immunofluorescence technique was conducted on the tight junction (TJ) protein. This study focused on ZO-1, a well-established junction protein that has been localized to these colonic cells. As shown in Figure 3, by the end of the 21 days of seeding, the monolayer of Caco-2 cells was perfectly formed, without the formation of a multilayer, which is considered one of the critical steps of the transwell assay method. Indeed, the formation of a monolayer of Caco-2 cells is important to reproduce the anatomical and functional characteristics of the intestinal epithelium. This is crucial to properly simulating the intestinal barrier, as multiple layers could alter the transport properties and interactions between cells.

#### 2.2.3. Lucifer Yellow Permeability Assay

The permeability of the Caco-2 cell monolayer was assessed using a lucifer yellow permeability assay, which determines the ability of luciferin yellow to cross the cell monolayer. After 4 h of treatment with the duodenal digested *S. lateriflora* extract, only 0.43 ± 0.16% of luciferin yellow crossed the cell monolayer. This result, which indicates the low permeability of luciferin through the cellular monolayer, confirms the integrity of the Ca-co-2 cell monolayer previously assessed with the TEER assay.

#### 2.2.4. Absorption Experiment on Caco-2 Cells Grown on Transwell Insert

**Figure 4:**
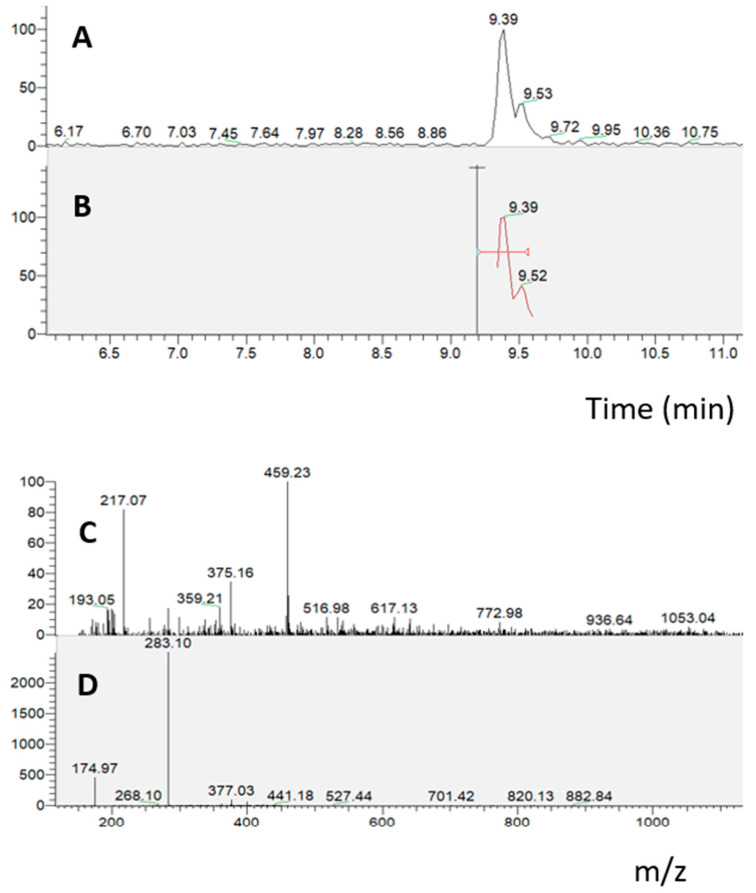
RP-UHPLC-PDA-ESI-MS/MS analysis of the solution taken from the basolater compartment, related to the absorption experiment on Caco-2 cells grown on a transwell insert treated with the duodenal digested *S. lateriflora* extract. (**A**,**B**) Mass chromatograms; (**C**,**D**) MS and MS/MS spectra of oroxylin A.

In the absorption experiment on the Caco-2 cell monolayer treated with the duodenal digested *S. lateriflora* extract (10 mg/mL), an RP-UHPLC-PDA-ESI-MS/MS analysis of the solution taken from the basolateral compartment revealed the presence of oroxylin A glucuronide, which presents an ion parent [M − H]^−^ at *m*/*z* 459 with a characteristic product ion at *m*/*z* 283, indicating that only oroxylin A glucuronide crossed the cell monolayer under the applied experimental conditions (Figure 4).

#### 2.2.5. Parallel Artificial Membrane Permeability Assay (PAMPA)

To conduct the bioavailability experiment using the PAMPA model system, a noncell-based assay for prediction of permeability that provides information about the passive permeability of a compound, solutions of caffeine and furosemide were used at different concentrations to evaluate the integrity of the artificial lipid membrane. The results presented in Table 3 demonstrate that, as expected, caffeine permeated the membrane. In contrast, furosemide did not cross the membrane, as indicated by the absorbance values obtained from spectrophotometric analyses of the acceptor compartment solutions. Thus, at the end of this preliminary test, the results showed that only caffeine was able to successfully cross the artificial lipid membrane (relative Log Pe: −1.83 cm^2^/s).

**Figure 5:**
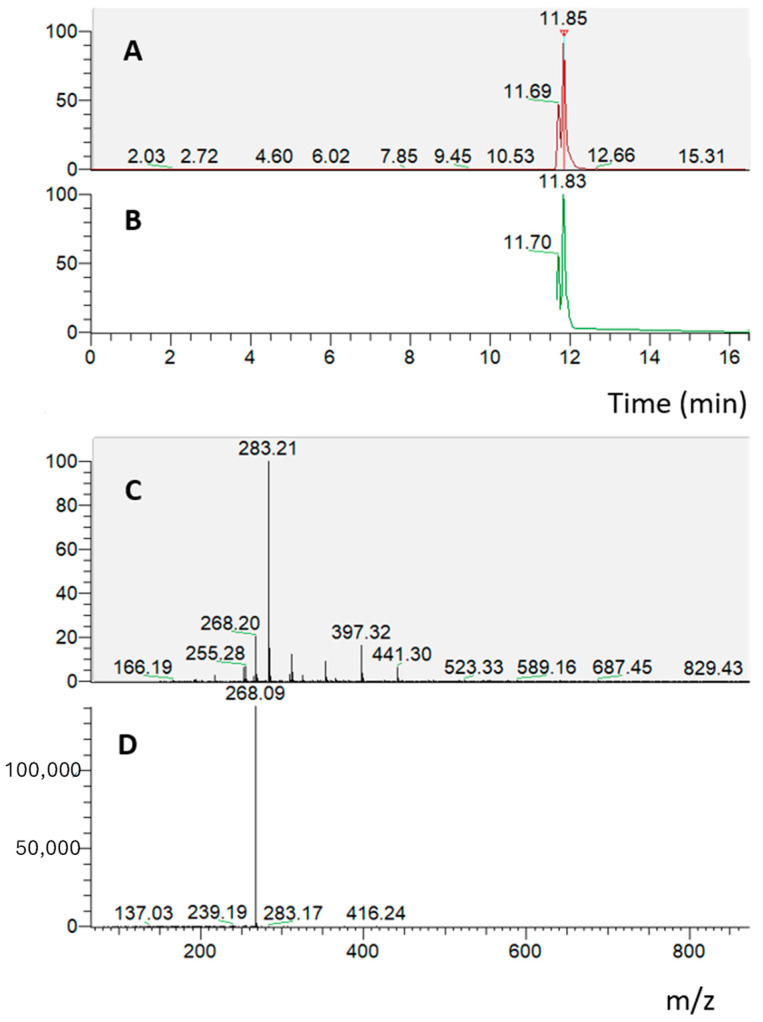
RP-UHPLC-PDA-ESI-MS/MS analysis of the solution taken from the basolater compartment, relating to the PAMPA experiment on the artificial lipid membrane treated with duodenal digested *S. lateriflora* extract. (**A**,**B**) Mass chromatograms; (**C**,**D**) MS and MS/MS spectra of oroxylin A.

Then, the duodenal digested *S. lateriflora* extract was submitted to a PAMPA assay. The solution taken from the basolateral compartment after 16 h of treatment at a concentration of 10 mg/mL was analyzed through the RP-UHPLC-PDA-ESI-MS/MS method. The chromatographic analysis revealed the presence of oroxylin A, which presents an ion parent [M − H]^−^ at *m*/*z* 283 with a characteristic product ion at *m*/*z* 268, indicating that only oroxylin A crossed the artificial lipid membrane under the applied experimental conditions (Figure 5).

### 2.3. Determination of Noncytotoxic Concentrations of S. lateriflora Extract in the H295R Cell Model System

**Figure 6:**
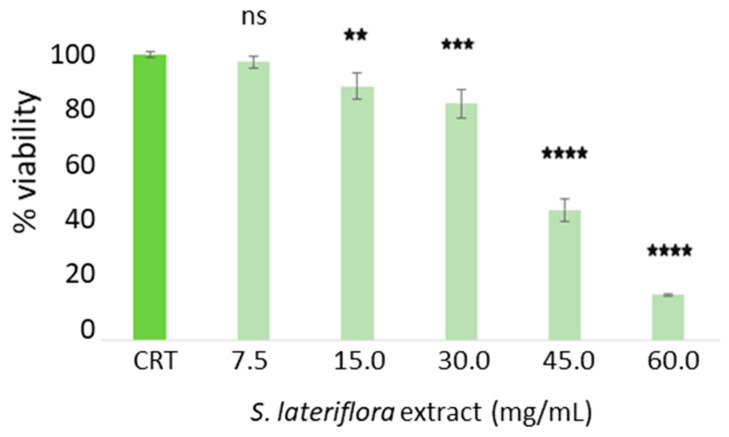
H295R Cell viability after treatment with *S. lateriflora* extract in the concentration range of 60–7.5 mg/mL. CTR: cells not treated with *S. lateriflora* extract. Data were expressed as mean ± SD from three biological replicates. ** *p* < 0.01, *** *p* < 0.001, **** *p* < 0.0001, and ns: not significant.

Prior to the determination of the potential ability of *S. lateriflora* extract to reduce or inhibit the release of cortisol, the noncytotoxic concentrations of *S. lateriflora* crude extract were determined 24 h post-treatment and compared with untreated control cells through an analysis of cell viability using the MTT assay. As shown in Figure 6, the treatment of H295R cells with increasing concentrations of *S. lateriflora* extract (7.5–60 mg/mL) led to a reduction in cell growth in a dose-dependent manner. Furthermore, the maximum noncytotoxic concentration, in which H295R cell viability exceeded 70%, was identified as 30 mg/mL, and this was utilized for the subsequent experiments.

### 2.4. In Vitro Inhibition of Cortisol Release by S. lateriflora Extract

**Figure 7:**
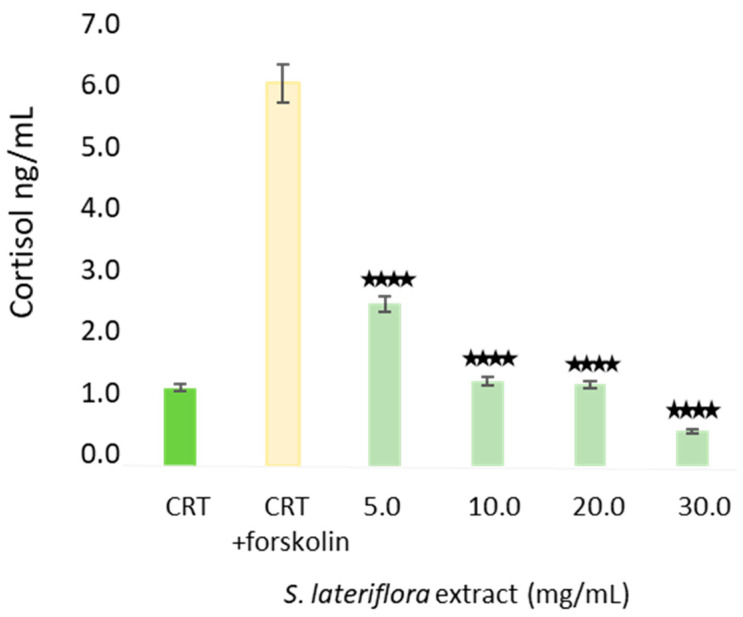
Concentration of cortisol (ng/mL) released from H295R cells in the basal state (CRT), following the treatment with forskolin (CRT + forskolin), and following the treatment of cells with increasing concentrations (5–30 mg/mL) of *S. lateriflora* extract. Data were expressed as mean ± SD from three biological replicates. **** *p* < 0.0001.

Figure 7 shows the concentrations of cortisol released by H295R human adrenocortical cells (1) in the basal state, (2) following the treatment with the adenylyl cyclase inducer forskolin in order to facilitate the detection of inhibitors, and (3) following the treatment with *S. lateriflora* extract at increasing noncytotoxic concentrations (i.e., 5, 10, 20, and 30 mg/mL). *S. lateriflora* extract reduced the percentage release of cortisol in a dose-dependent manner ranging from 58 to 91%, with *S. lateriflora* extract concentrations of 30 mg/mL being the most active treatment (*p* < 0.0001).

## 3. Discussion

In this investigation, a commercial extract rich in polyphenols obtained from the aerial parts of *S. lateriflora,* previously characterized using ultra-high-performance liquid chromatography (UHPLC) coupled with a Q-Exactive hybrid quadrupole orbitrap mass spectrometer [3] was submitted to in vitro studies, including the determination of bioaccessibility, bioavailability, and investigation of cortisol release inhibition as a possible mechanism of action through which *S. lateriflora* exerts its protective effects against the numerous health concerns including psychiatric issues (i.e., stress, insomnia, anxiety, and depression) and nonpsychiatric issues (i.e., osteopenia and osteoporosis) associated to an excess in cortisol release.

In vitro simulated gastric, duodenal, and gastroduodenal digestion processes revealed a significant reduction in the peak area of some of the most represented polyphenolic compounds, including flavones (apigenin derivative, scutellarin, baicalein 6-glucuronide, and oroxilyn A glucuronide), a flavanone (naringenin 7-*O*-d-hexoside 6″ acetate), and an isoflavone (genistein). Postdigestion, greater degradation was observed for scutellarin and genistein, while the degradation was less pronounced for the other compounds.

The bioavailability assays (Caco-2 cells grown on transwell insert assay and PAMPA assay) showed oroxylin A glucuronide and oroxylin A as the compounds of interest, as they were the only compounds that were able to cross the Caco-2 cell membrane and the artificial lipid membrane, respectively. It is interesting that, in agreement with hydrophilic and lipophilic features, oroxylin A, which is more liposoluble than its glucuronide form, crosses the artificial lipid membrane, while the glucuronide form crosses the cell monolayer.

Extensive literature data indicates a considerable reduction in the polyphenolic content of vegetable extracts [19,20,21] after gastroduodenal digestion. None of the previous studies reported the bioaccessibility of *S. lateriflora* extracts, and this is the first study to report the bioaccessibility of the main polyphenolics of *S. lateriflora* extract following gastroduodenal digestion. A study by Ullah et al. [3] on the in vitro bioaccessibility of a *S. lateriflora* extract after oral digestion showed a modest reduction in the concentrations of the main components of the extract, with a greater reduction in the chromatographic peak area of scutellarin (8.9%). While assessing the in vitro bioaccessibility of *Eugenia pyriformis* Cambess. fruit, De Paulo Farias et al. [22] observed a significant reduction in the total flavonoid contents (including apigenin hexoside) following gastric (48%) and intestinal (70%) digestion. Conversely, our results appear to contradict those reported by Walsh et al. [23], which demonstrated the good bioaccessibility of isoflavones due to their higher stability under gastroduodenal digestion conditions, although the authors concluded that this good stability of phenolic compounds may be attributed to an increased secretion of bile acid stimulated by the presence of proteins and fats in food samples. Baicalein has been reported as having poor water solubility (16.82 μg/mL) that reduces its bioaccessibility and, in turn, limits the bioavailability of the compound [24,25]. It has also been documented that polyphenols are highly sensitive to the mild alkaline conditions in the small intestine, where degradation of most polyphenolic compounds takes place or, in other cases, transformation into other metabolites occurs [26].

Prior to the assessment of the bioavailability of *S. lateriflora* extract components, the noncytotoxic concentration of *S. lateriflora* extract, corresponding to a cell viability higher than 70%, was evaluated by MTT assay and was found to be 30 mg/mL. Our results, showing the low bioavailability of the polyphenolic compounds occurring in *S. lateriflora* extract, are in agreement with the available literature, where low bioavailability of flavonoids (i.e., apigenin, scutellarein, genistein, baicalein, quercetin, and naringenin) is reported across the studies [26,27,28,29,30,31,32]. The main properties limiting the bioavailability of polyphenols are their poor water solubility and low potential to penetrate the intestinal membrane, as reported in the case of scutellarin [33,34], which has a water solubility of only approximately 14–20 µg/mL and does not cross the intestinal membrane. Another factor that might contribute to the low bioavailability of polyphenols is their strong association with cell walls, which may result in their retention in the nondigested fraction [35]. Moreover, in the present study, *S. lateriflora* extract was subjected only to simulate in vitro duodenal digestion before the bioavailability assays, without fermentation with gut microbiota. This could be a limitation, as most flavonoids generally require metabolic transformation through the intestinal microflora prior to absorption through the intestinal cell membrane [36]. Although it is difficult to compare data obtained from in vitro tests with data obtained in vivo on experimental animals, however the in vivo pharmacokinetics analysis of Scutellariae radix, with the determination of baicalin, wogonoside, and oroxylin, indicated the presence of all these flavonoids in blood samples in their glycoside and aglycone forms after oral administration of rats with this plant extract [37]. Partially in agreement with our study, oroxylin A glucuronide was the glycoside present in the highest concentration, while baicalein was the flavonoid with the poorest intestinal permeability as both glucuronide and aglycone. Another study showed the rapid absorption, tissue distribution, and elimination of oroxylin A aglycone and glucuronide after oral administration [38].

Oroxylin A is a flavone, containing one methoxy group at carbon 6 and two hydroxyl (OH) groups at carbons 5 and 7. Oroxylin A 7-*O*-β-d-glucuronide is one of the principle metabolites of oroxylin A, and is a monomethoxy and dihydroxy flavone derivative of the parent compound [39]. Based on its chemistry, oroxylin A could be a flavonoid with strong bioactivity. Indeed, some studies have revealed a wide spectrum of biological activities of oroxylin A, such as anti-inflammatory, anticancer, anti-invasive, neuroprotective, and antiangiogenic effects [40]. However, the cortisol inhibition effects of oroxylin A have not been reported to date, but the potential neurological effects (i.e., dopamine reuptake inhibition, regulation of brain-derived neurotrophic factor (BDNF) expression, improved memory and cognitive functions, neuroprotective actions via decreasing markers for neuroinflammation, improvement of the attention-deficit hyperactivity disorder (ADHD)-like behaviors) do indicate its activity within the nervous system [40]. A study showed beneficial effects against chronic stress through the regulation of hippocampal BDNF expression and neurogenesis in C57BL/6J mice [41].

Prior to the assessment of its in vitro inhibition of cortisol release, the noncytotoxic concentration of *S. lateriflora* extract, corresponding to a cell viability higher than 70%, was evaluated by MTT assay and was found to be 30 mg/mL. The treatment of H295R cells with noncytotoxic concentrations of *S. lateriflora* extract showed a significant inhibition of cortisol release for all tested doses in a dose-dependent manner, with 30 mg/mL being the most potent concentration, resulting in 91% of cortisol inhibition. Increasing evidence suggests the potential link between circulating cortisol levels and stress-related disorders, including depression and anxiety, as stress disorders are linked with the activity of the HPA axis. In early investigations, patients with coexisting panic disorders, depression, and agoraphobia were found to have significantly elevated urinary cortisol levels compared to healthy individuals, and benzodiazepines were found to be effective in decreasing these free cortisol levels [42]. Another study showed elevated nocturnal levels of stress hormones, including cortisol, epinephrine, and norepinephrine, in subjects with panic disorders [43]. An investigation by Vedhara et al. [44] exhibited a nonlinear relationship between cortisol levels and the time of day, where this nonlinear phenomenon was largely based on stress and anxiety levels. The study determined a change in cortisol levels at specific times of day, but changes in absolute levels of the hormone were not observed. Vreeburg et al. [45] demonstrated significantly higher salivary levels of cortisol early in the morning (i.e., 1 h cortisol awakening response) in patients with panic disorders comorbid with agoraphobia and depression.

This work has its limitations and strengths. The main limitation of this study is the exclusion of fermentation by the gut microbiota, due to which the potential impact of the gut microbiome on the bioaccessibility and bioavailability of polyphenolic compounds remains undetermined. Another limitation of the present study is that it does not explore the inhibition of cortisol release at the molecular level. Hence, this study could be considered a preliminary evaluation, contributing scientific evidence to the theory that the relaxation properties of the *S. lateriflora* extract may be justified by inhibition of cortisol release. The major strength of this study is that the property of *S. lateriflora* extracts to the inhibit cortisol release is determined for the first time, suggesting a possible mechanism of action behind the relaxation activity traditionally ascribed to *S. lateriflora*. Another strength of this study is that the bioaccessibility and bioavailability of *S. lateriflora* extract were investigated for the first time, providing a picture of the bioavailable compounds and suggesting the need for gastroprotective dosage forms. 

In conclusion, the *S. lateriflora* extract could be a good agent against the cortisol-mediated stress response and associated pathologies. This effect could also be useful for counteracting bone loss and osteoporosis induced by high cortisol levels, since, as previously mentioned, cortisol levels and bone density are frequently linked, especially in high-risk and postmenopausal women. However, the low bioaccessibility of the *S. lateriflora* extract suggests the use of a gastroprotectant formulation for the possible applications of this extract as a food supplement ingredient for patients complaining of insomnia and sleep disorders. A clinical trial assessing the efficacy and tolerability of a *S. lateriflora* extract-based food supplement for maintaining a correct sleep-wake cycle is currently in progress.

## 4. Materials and Methods

### 4.1. Chemicals, Reagents and Biological Materials

Caco-2 and H295R cells were purchased from the American Type Culture Collection (ATCC CRL-2128™; distribution by LGC group, London, UK). The cell culture medium, fetal bovine serum (FBS), nonessential amino acids and lucifer yellow (LY) were bought from M & M Biotech (Naples, Italy). All other cell culture reagents were purchased from Sigma-Aldrich (St. Louis, MO, USA). Finally, transwell PET inserts were obtained from Sterlitech Corporation (Washington, DC, USA). Human COR ELISA Kit (3455-HP-2) was bought from FineTest^®^ (Prodotti Gianni S.r.l., Milan, Italy).

Three batches of commercial, dry, powdered hydroalcoholic extract of *S. lateriflora* (standardized to contain ≥10% of baicalin, and maize maltodextrin as a carrier agent) obtained from the aerial parts of the plants, were provided by EPO S.R.L. (Milan, Italy). All the compounds used for in vitro gastric and duodenal digestion processes have been reported as follows: potassium chloride (KCl), dihydrogen potassium phosphate (KH_2_PO_4_), sodium carbonate (NaHCO_3_), magnesium chloride (MgCl_2_), ammonium carbonate (NH_4_)_2_CO_3_, calcium chloride (CaCl_2_), sodium chloride (NaCl), hydrochloric acid (HCl), sodium hydroxide (NaOH). All compounds were provided by Carlo Erba (Milan, Italy). Pancreatin from a porcine pancreas (extract of pig bile), pepsin from porcine gastric mucosa and porcine bile extract, formic acid solution (1 M), water, methanol, acetonitrile LC-MS grade, and dimethyl sulfoxide (DMSO) were purchased from Sigma-Aldrich, Merck KGaA (Milan, Italy).

### 4.2. In Vitro Bioaccessibility of S. lateriflora Extract

In vitro digestion of *S. lateriflora* extract was performed by simulated gastric, duodenal, and gastroduodenal digestion processes following protocols set by Minekus et al. with slight modifications [46].

#### 4.2.1. In Vitro Simulated Gastric Digestion

To evaluate the effect of the gastric digestion process on *S. lateriflora* extract, 1.0 g of sample was dissolved in 2 mL of bidistilled water. Simulated gastric fluid (SGF) electrolyte stock solution (1.5 mL) was then added, followed by 0.32 mL porcine pepsin stock solution (25,000 U/mL prepared in SGF) and 1 μL of CaCl_2_ (0.3 M). Finally, 0.2 mL of HCl (1 M) was added to achieve pH 3.0 and made up with bidistilled water to obtain a final volume of 4 mL. The process was repeated, replacing the sample with a blank consisting of 2 mL of bidistilled water. The reaction vessels were placed onto a shaking platform at 37 °C for 2 h. At the end of the digestion process, the samples were kept at −20 °C prior to RP-UHPLC-PDA-ESI-MS/MS analysis.

#### 4.2.2. In Vitro Simulated Duodenal Digestion

Similarly, as described before, 1.0 g of sample was dissolved in 4 mL of bidistilled water, mixed with 2.2 mL of simulated intestinal fluid (SIF) electrolyte stock solution, 1 mL of a pancreatin solution (800 U/mL prepared in SIF electrolyte stock solution), 0.5 mL fresh bile (160 mM in fresh bile) and 8 μL of CaCl_2_ (0.3 M). Once the reaction tube was prepared, 0.15 mL of NaOH (1 M) was added to reach pH 7.0 with bidistilled water to obtain a final volume of 8 mL. Here too, the process was repeated in order to have a negative control. Finally, the reaction vessels were placed onto a shaking platform at 37 °C for 2 h. At the end of the digestion process, the samples were maintained at −20 °C prior to RP-UHPLC-PDA-ESI-MS/MS analysis.

#### 4.2.3. In Vitro Simulated Gastroduodenal Digestion

To assess the effect of the full gastroduodenal process on *S. lateriflora* extract, 1.0 g of sample was dissolved in 2 mL of bidistilled water, and 0.3 mL of SGF electrolyte stock solution was then added, followed by 0.064 mL porcine pepsin stock solution (25,000 U/mL prepared in SGF) and 1 μL of CaCl_2_ (0.3 M). Subsequently, 0.2 mL of HCl (1 M) was added to reach a solution of pH 3.0 with bidistilled water to obtain a total volume of 4 mL. A 4 mL blank sample of bidistilled water was put through the same process. The reaction vessels were then placed onto a shaking platform at 37 °C for 2 h. Then, the simulated gastric samples were added to 2.2 mL of SIF electrolyte stock solution, 1 mL of a pancreatin solution (800 U/mL prepared in SIF electrolyte stock solution), 0.5 mL fresh bile (160 mM in fresh bile), and 8 μL of CaCl_2_ (0.3 M). Moreover, 0.15 mL of NaOH (1 M) were added to reach pH 7.0, with bidistilled water to reach the final volume of 8 mL. Finally, the reaction vessels were placed onto a shaking platform at 37 °C for 2 h. At the end of the digestion process, the samples were maintained at −20 °C prior to RP-UHPLC-PDA-ESI-MS/MS analysis.

#### 4.2.4. RP-UHPLC-PDA-ESI-MS/MS Analysis

RP-UHPLC-PDA-ESI-MS/MS analyses were performed using an Ultimate 3000 SD UHPLC system, equipped with a quaternary pump, autosampler, and column heater compartment. The chromatographic system was connected to a Surveyor UV–Vis photodiode array detector (PDA), using an ESI source at an LTQ XL Linear Ion Trap Mass Spectrometer (Thermo Fischer Scientific, Waltham, MA, USA). For UHPLC analysis a Kinetex^®^ EVOTM 150 mm × 2.1 mm, 2.6 µm (L × I.D, particle size, Phenomenex^®^, Bologna, Italy) column was employed at a flow rate of 0.4 mL/min. The mobile phases consisted of (A) 0.1% CH_3_COOH in H_2_O and (B) can plus 0.1% CH_3_COOH. Analysis was performed in gradient as follows: 0–10.0 min, 2–35% B; 10–12 min, 35–50% B; 12–13 min, 50–60%; 13–25 min, 70–75% B; 25–30 min, 75–95% B; 30–31 min, 95–5% B; 31–32 min, 95–2% B; hold for 2 min; return to initial conditions in 0.1 min. Column oven was set to 40 °C, and 5 µL of extracts were injected. Chromatogram measurements were taken at 254, 280, and 324 nm; spectral data were taken between 200 and 800 nm for every peak. HRMS analysis was performed with a full MS scan (*m*/*z* 100–850) and data-dependent acquisition (dd-MS2 top N = 5). A resolution of 70,000 and 15,000 FWHM at *m*/*z* 200 was selected. A collision energy (NCE) value of 35 were used. Negative ESI was employed. Aliquots of 10 mg of freeze-dried digested samples were dissolved in 2 mL of H_2_O/MeOH: 50/50 prior to chromatographic analysis.

### 4.3. In Vitro Bioavailability

#### 4.3.1. Cell Culture

Human colon carcinoma Caco-2 (ATCC, HTB-37tm), from the American Type Culture Collection, was cultivated in Dulbecco’s modified Eagle’s medium (DMEM) media supplemented with 10% fetal bovine serum (FBS), 1% nonessential amino acids (Gibco), 100 U/mL penicillin, and 100 μg/mL streptomycin. The cells were kept at 37 °C in a 5% CO_2_ incubator and the complete media was replaced every 2 days before reaching 90% confluence.

#### 4.3.2. Cytotoxic Activity of Duodenal Digested *S. lateriflora* Extract on Caco-2 Cells

The cell viability assay (MTT test) was also performed on the Caco-2 cell line, to determine the maximum noncytotoxic concentration of duodenal digested *S. lateriflora* extract. The MTT assay measures cellular metabolic activity as an indicator of vitality, proliferation, and cellular cytotoxicity [47]. Cells were cultured in DMEM and supplemented with 1% penicillin-streptomycin and 10% fetal bovine serum, at 37 °C with 5% CO_2_ in a humid environment, as previously described. A density of 3 *×* 10^4^ cells/well was seeded into 96-well plates, and cells were cultured at 37 °C with 5% CO_2_ in a humid environment for 4 h, as previously described. After 4 h of incubation, the cells were treated with different concentrations of duodenal digested *S. lateriflora* extract (60–7.5 mg/mL). After the incubation period, the culture medium was removed from each well and the cells rinsed with phosphate-buffered saline (PBS) before adding the 3-(4,5-dimethylthiazol-2-yl)-2,5-diphenyltetrazolium bromide (MTT, Sigma-Aldrich, St. Louis, MO, USA) at a final concentration of 0.5 mg/mL. The cells were incubated for 3 h at 37 °C. The formazane crystals were then solubilized with 100 μL DMSO/well for 10 min and the viability rate was recorded at OD at 570 nm using a Multiskan™ GO microplate reader (Thermo Scientific Microplate Spectrophotometer, Thermo Fisher Scientific, Waltham, MA, USA). The experiment was performed in triplicate.

#### 4.3.3. PAMPA Assay

The PAMPA assay is a noncell-based assay, based on the ability of the compound to diffuse from a donor compartment into an acceptor compartment, through a PVDF membrane filter, pretreated with a lipid solution dissolved in organic solvent. The test was conducted following the Manufacturer’s instructions (MultiScreen Permeability Plates from MilliPore Sigma, Burlington, MA, USA). Therefore, a 1% solution (*w*/*v*) of lecithin in dodecane was prepared to mimic the phospholipid membrane and sonicated until completely dissolved. *S. lateriflora* samples were prepared and 1 mg of the duodenal digested sample was weighed and resuspended in 1 mL of PBS 5% DMSO pH 7.4 (buffer solution) to obtain a stock solution at a final concentration of 10 mg/mL. Finally, each test used a positive control with high permeability (caffeine) and a negative control with low permeability (furosemide) as according to the Biopharmaceutical Classification System (BCS), both solubilized in the same conditions of the duodenal digested *S. lateriflora* extract. An aliquot of a 5 uL lecithin/dodecane mixture was carefully pipetted into each Donor plate. Next, 300 μL of PBS 5% DMSO was added to each well of the Acceptor plate, and 150 μL of the duodenal digested *S. lateriflora* samples were added into each well of the Donor compartment. After that, the Donor and Acceptor plates were assembled, making sure the underside of the membrane was in contact with the buffer solution, and incubated at room temperature for 16 h. At the end of the incubation period, 100 μL/well from the Donor compartment and 250 μL from the Acceptor compartment were taken to measure UV/Vis absorption from 250 to 500 nm. In addition, to calculate the log P_e_ (log of the effective permeability of the compounds) as follows below, the theoretical equilibrium concentrations for each compound (and relative concentration tested) were measured.

$$Log\;P_{e\;}=\left\{C*-Ln\;(1-\frac{{\left[drug\right]}_{acceptor}}{{\left[drug\right]}_{equilibrium}})\right\}$$

$$C=\left(\frac{Vd*Va}{\left(Vd+Va\right)Area*Time}\right)$$

To evaluate membrane integrity, calibration curves for caffeine and furosemide were prepared at concentrations ranging from 500 to 7.81 μM for the caffeine solution and from 500 to 31.25 μM for the furosemide solution. Spectrophotometric analysis was perfomed using a multiplate reader (Perkin Elmer, Waltham, MA, USA) and a 96-well quartz plate (Hellma Analytics, Müllheim, Germany).

#### 4.3.4. Caco-2 Transwell Model System

For the transport experiments, the protocol according to Husbatrsch et al. [48] was followed, with some modifications. Caco-2 cells were seeded at a density of 3 × 10^5^ cells/well on transwell PET inserts (0.4 μm pore size, CellQART^®^-Sterlitech, Auburn, WA, USA) and cultured in complete DMEM medium for 21 days, with the medium being changed three times a week. The transport experiment was performed in the apical-to-basolateral direction, with 0.5 mL of medium in the apical compartment and 1.5 mL medium in the basolateral compartment. Before carrying out the experiments, the monolayer integrity was evaluated by measuring the trans-epithelial electrical resistance (TEER) with an EVOM2™ Epithelial Voltohmmeter (World Precision Instrument, Sarasota, FL, USA). TEER was measured at days 7, 15 and 21 and TEER was measured after subtraction of the intrinsic resistance of the cell free insert according with the following formula:

$$R_{Tissue}\left(\Omega \right)=culture\;insert\;value-control$$

$$TEERf=R_{Tissue}\;\times \;\Omega {cm}^{2}$$

Moreover, once the permeability test was completed, the permeability of a paracellular marker, LY (Invitrogen, Waltham, MA, USA, Lucifer Yellow CH, St. Louis, MO, USA, Lithium Salt, Qinghai, China), was assessed by analyzing the fluorescence using a fluorescence microplate reader (Envision microplate reader, Perkin Elmer, Waltham, MA, USA) at an excitation wavelength of 425 nm and an emission wavelength of 528 nm. The confluent monolayers were then used for further experiments.

Prior to the permeability assay, the inserts were washed twice and equilibrated for 30 min with prewarmed transport medium, Hank’s balanced salt solution (HBSS), pH 7.4. Thus, cells were treated with the duodenal digested *S. lateriflora* extract at a final concentration of 10 mg/mL and the transwell plates were incubated at 37 °C under agitation (VWR^®^ Microplate Mixer, Radnor, PA, USA). After 30, 60, 90, 150 and 210 min, aliquots (200 μL) were collected from the acceptor compartment and replaced with the same volume of prewarmed buffered HBSS to maintain sink conditions. Finally, whole medium was collected from the donor compartment and submitted to the chromatographic analysis.

#### 4.3.5. Immunochemistry

After 21 days culture, the medium in the 12-well transwell chamber was exhausted, and cells were washed three times with 500 μL PBS for 5 min each. Then, 500 μL paraformaldehyde 4% was added to each chamber, and the cells were fixed for 10 min. After permeabilization for 10 min with 0.25% Triton X-100 (in PBS), cells were blocked with 5% BSA in PBS for 1 h at room temperature. A working solution of ZO-1/TJP1 Antibody, Alexa Fluor^®^ 594 conjugate (ZO1-1A12), was prepared at a concentration of 5 µg/mL in 1% BSA from a more concentrated stock solution. Subsequently, cells were labelled and incubated for 3 h at room temperature. Afterward, DAPI (Invitrogen) was used for counterstaining of the nucleus. Finally, each insert membrane was cut out, placed between two cover slips, and analyzed using a fluorescent optical microscope (DM2000 LED-Leica, Leica Microsystems, Wetzlar, Germany).

### 4.4. In Vitro Efficacy of S. lateriflora Extract on the Release of Cortisol

#### 4.4.1. Cell Culture

H295R cells were maintained in Dulbecco’s modified Eagle’s medium/Hams’s F-12 (DMEM-F12) supplemented with 5% fetal bovine serum (FBS), insulin, apotransferrin, selenium and antibiotic (penicillin/streptomycin 100 g/mL). An atmosphere of 5% CO_2_ at 37 °C was provided and the medium was replaced every 48 h.

#### 4.4.2. Cytotoxic Activity of *S. lateriflora* Extract on H295R Cells

To assess the maximum noncytotoxic concentration of *S. lateriflora* extract on the H295R cell line, a 3-(4,5-dimethylthiazol-2-yl)-2,5-iphenyltetrazolium bromide (MTT) assay was performed. The MTT assay measures cellular metabolic activity as an indicator of vitality, proliferation, and cellular cytotoxicity [47]. Cells were cultured in DMEM-F12 and supplemented with 5% fetal bovine serum, insulin, apotransferrin, selenium and 1% penicillin-streptomycin, at 37 °C, with 5% CO_2_ in a humid environment, as previously described.

Thus, a density of 5 × 10^4^ cells/well was seeded into 96-well plates and cells cultured at 37 °C with 5% CO_2_ in a humid environment for 24 h. The cells were treated with different concentrations of *S. lateriflora* extract (7.5–60.0 mg/mL). After the incubation period, the cells were treated with the 3-(4,5-dimethylthiazol-2-yl)-2,5-diphenyltetrazolium bromide (MTT, Sigma-Aldrich, St. Louis, MO, USA) with a final concentration of 0.5 mg/mL. The cells were incubated for 3 h at 37 °C. The formazane crystals were then solubilized with 100 μL DMSO/well for 10 min, and the viability rate was recorded at OD at 570 nm using a Multiskan™ GO microplate reader (Thermo Scientific Microplate Spectrophotometer). The current experiment was performed in triplicate.

#### 4.4.3. In Vitro Evaluation of the *S. lateriflora* Extract on Inhibition of CORTISOL Release

In order to evaluate the effects of *S. lateriflora* extract on the release of cortisol, in vitro experiments were carried out by treating the cells with forskolin (20 μmol/L) to stimulate the production of cAMP and therefore steroidogenesis. Cells were seeded in sterile 6-multiwell plates in DMEM-F12 medium, supplemented with 5% FBS at a density of 4 × 10^5^ cells. The cells were left in the incubator for 48 h. Subsequently, cells were harvested using low serum concentration medium (0.1% FBS) and the cells were incubated overnight at 37 °C with 5% CO_2_, followed by replacement of the test medium containing 0.1% FBS, varying *S. lateriflora* extract treatment concentrations (5.0–30.0 mg/mL) and forskolin. After 24 h of incubation, the cell culture medium was collected, and the cortisol concentration was analyzed through ELISA assay in accordance with the manufacturer’s instructions. The treatment and the determination of cortisol were performed in triplicate.

#### 4.4.4. Evaluation of Cortisol Levels by ELISA Assay

The concentration of cortisol in H295R cell culture supernatant samples was evaluated using the enzyme-linked immunosorbent assay (ELISA) with commercially available test kits. Samples were diluted where necessary; the standards of known concentration and the blanks were prepared for the elaboration of calibration lines. Samples and standards were incubated together with the primary antibody, and then with the secondary antibody in multiwell plates. Finally, the peroxidase enzyme substrate conjugated to the secondary antibody was added to develop a colored product. The absorbance values are related to the cortisol concentration according to an exponential trend. The absorbance was recorded by spectrophotometer at a wavelength of 450 nm. The data obtained were processed with the MyAssay software (https://www.myassays.com/index.html, accessed on 22 January 2024).

### 4.5. Statistical Analysis

For the MTT assay performed on the H295R cell line and the Caco2 cell line, the concentrations of cortisol tested with the ELISA assay, and the percentages of degradation of the *S. lateriflora* extract components analyzed by RP-UHPLC-PDA-ESI-MS/MS, data were expressed as mean ± standard deviation (SD) from three biological replicates.

Concerning the viability percentages of the H295R cell line and the Caco2 cell line, and the concentrations of cortisol, the statistical comparison among groups was conducted using one-way ANOVA followed by Dunnett’s post hoc test for multiple comparisons to determine significance, which was set to *p* < 0.05. The statistical analyses were performed using GraphPad Prism, version 8 (San Diego, CA, USA).

## Figures and Tables

**Table 1:** Mean peak area of six compounds identified in *S. lateriflora* extract before (UND) and after gastric (GD), duodenal (DD), and gastroduodenal digestion (GDD) and area reduction percentage (%).

Sample	RT [min]	Mean Area	Area Reduction Percentage (%)
UND	8.08	5.62 × 10^4^	
	8.16	1.55 × 10^4^	
	8.77	1.17 × 10^6^	
	9.06	1.28 × 10^5^	
	9.26	7.50 × 10^4^	
	10.44	4.70 × 10^4^	
GD	8.08	3.64 × 10^4^	35.3
	8.17	9.70 × 10^3^	37.2
	8.77	8.67 × 10^5^	25.6
	9.06	8.74 × 10^4^	31.6
	9.26	5.92 × 104	21.3
	10.44	2.09 × 104	55.7
DD	8.08	3.63 × 104	35.6
	8.17	8.58 × 10^3^	44.5
	8.77	8.64 × 10^5^	25.9
	9.05	8.92 × 10^4^	30.2
	9.25	5.97 × 10^4^	20.7
	10.44	1.59 × 10^4^	66.3
GDD	8.07	2.63 × 10^5^	53.2
	8.16	5.93 × 10^3^	61.6
	8.76	7.63 × 10^5^	34.7
	9.05	7.82 × 10^4^	38.9
	9.25	4.93 × 10^4^	34.3
	10.44	1.27 × 10^4^	73.2

**Table 2:** TEER values of the Caco-2 culture cell model after 21 days of incubation. The blank corresponds to the TEER value measured for an insert containing HBSS but devoid of cells. As per the protocol, this value has been subtracted from the TEER values obtained for each sample to normalize the data.

Sample	TEER Ω (Sample Value)	TEER Ω Corrected
S1	1625	1510
S2	1720	1605
S3	1780	1665
SD1	1785	1670
SD2	1760	1645
SD3	1768	1653
BLANK	115	0

**Table 3:** Results of the PAMPA experiment evaluating the permeability of caffeine and furosemide at different concentrations.

Concentration	OD 1	OD 2	OD 3	Mean Abs
Caffein 500 μM	0.839	0.938	0.961	0.913
Caffein 250 μM	0.528	0.504	0.526	0.519
Caffein 125 μM	0.277	0.264	0.291	0.277
Caffein 62.5 μM	0.152	0.145	0.153	0.150
Caffein 31.25 μM	0.081	0.088	0.105	0.091
Caffein 15.62 μM	0.059	0.054	0.055	0.056
Caffein 7.81 μM	0.035	0.040	0.045	0.040
Furosemide 500 μM	0.010	0.010	0.010	0.010
Furosemide 250 μM	0.024	0.030	0.022	0.025
Furosemide 125 μM	0.018	0.019	0.019	0.019
Furosemide 125 μM	0.031	0.021	0.029	0.027
Furosemide 62.5 μM	0.021	0.021	0.020	0.021
Furosemide 31.25 μM	0.033	0.018	0.020	0.024
Blank				0.010

## Data Availability

The data presented in this study are available in the article.
